# Thrombus composition and distribution patterns by thrombus volume in acute ischemic stroke

**DOI:** 10.3389/fneur.2025.1619683

**Published:** 2025-08-04

**Authors:** Jaeseob Yun, Jae Wook Jung, Kwang Hyun Kim, Hyo Suk Nam, JoonNyung Heo, Hyungwoo Lee, Byung Moon Kim, Dong Joon Kim, Minyoul Baik, Joonsang Yoo, Jinkwon Kim, Hyungjong Park, Sung-Il Sohn, Jeong-Ho Hong, Tae-Jin Song, Yoonkyung Chang, Jung Hwa Seo, Gyu Sik Kim, Kwon-Duk Seo, Seong Hwan Ahn, Jang-Hyun Baek, Han-Jin Cho, Jin Kyo Choi, Yo Han Jung, Bang-Hoon Cho, Il Kwon, Ji Hoe Heo, Young Dae Kim

**Affiliations:** ^1^Department of Neurology, Keimyung University School of Medicine, Daegu, Republic of Korea; ^2^Department of Neurology, Yonsei University College of Medicine, Seoul, Republic of Korea; ^3^Department of Neurology, Kyungpook National University Chilgok Hospital, School of Medicine, Kyungpook National University, Daegu, Republic of Korea; ^4^Integrative Research Institute for Cerebrovascular and Cardiovascular Diseases, Yonsei University College of Medicine, Seoul, Republic of Korea; ^5^Department of Radiology, Yonsei University College of Medicine, Seoul, Republic of Korea; ^6^Department of Neurology, Yongin Severance Hospital, Yonsei University College of Medicine, Yongin, Republic of Korea; ^7^Department of Neurology, Seoul Hospital, Ewha Woman’s University College of Medicine, Seoul, Republic of Korea; ^8^Department of Neurology, Mokdong Hospital, Ewha Woman’s University College of Medicine, Seoul, Republic of Korea; ^9^Department of Neurology, Busan Paik Hospital, Inje University College of Medicine, Busan, Republic of Korea; ^10^Department of Neurology, National Health Insurance Service Ilsan Hospital, Goyang, Republic of Korea; ^11^Department of Neurology, Chosun University School of Medicine, Gwangju, Republic of Korea; ^12^Department of Neurology, Kangbuk Samsung Hospital, Sungkyunkwan University School of Medicine, Seoul, Republic of Korea; ^13^Department of Neurology, Pusan National University School of Medicine, Busan, Republic of Korea; ^14^Department of Neurology, Seoul Medical Center, Seoul, Republic of Korea; ^15^Department of Neurology, Gangnam Severance Hospital, Yonsei University College of Medicine, Seoul, Republic of Korea; ^16^Department of Neurology, Korea University Anam Hospital and College of Medicine, Seoul, Republic of Korea; ^17^Department of Neurology, CHA Bundang Medical Center, Seongnam, Republic of Korea

**Keywords:** thrombus, ischemic stroke, thrombus volume, histology, thrombectomy

## Abstract

**Background:**

Thrombus burden considerably impacts ischemic stroke presentation and outcomes. However, the relationship between thrombus histology and volume has not been studied well. We investigated whether ischemic stroke thrombus composition and spatial distribution patterns differed with thrombus volume.

**Methods:**

We enrolled patients with thrombi undergoing endovascular therapy (EVT) between July 2017 and July 2023. Thrombus volume on thin-section non-contrast computed tomography was measured using three-dimensional software. Immunohistochemistry analysis included fibrin, red blood cells (RBCs), and platelets. Thrombi were classified based on the overall distribution pattern of the components: layered, erythrocytic, mixed, and diffuse platelet. We analyzed the association between thrombus volume, histopathology, distribution patterns, and clinical/radiologic outcomes.

**Results:**

Among 210 patients, the median (interquartile range) thrombus volume was 43.7 (23.5, 74.5) mm^3^. Increased thrombus volume correlated with high RBC proportion (*r* = 0.359, *p* < 0.001) and low platelet proportion (*r* = −0.194, *p* = 0.005). Thrombus volume was independently related to the RBC proportion (*β* 1.00, standard error [SE] 0.27, *p* < 0.001), mixed (*β* 21.04, SE 10.10, *p* = 0.038), and erythrocytic pattern (*β* − 29.78, SE 11.54, *p* = 0.011). The number of fragmented thrombi during the procedure was independently related to thrombus volume (*β* 0.006, SE 0.002, *p* = 0.006) and RBC proportions (*β* 0.18, SE 0.009, *p* = 0.049).

**Conclusion:**

Large thrombi had increased RBC proportions and a mixed pattern. RBC incorporation significantly contributes to the volumetric growth of thrombi and their fragmentation susceptibility. These findings may provide additional clue for tailoring EVT strategies.

## 1 Introduction

Thrombotic diseases, including ischemic stroke, significantly contribute to morbidity and mortality worldwide (1). Acute ischemic stroke caused by large vessel occlusion (LVO) is commonly treated with endovascular therapy (EVT), during which thrombi are extracted, providing opportunities for detailed analyses of thrombus histology (2). Such studies are important for elucidating thrombus composition, mechanisms of thrombus formation which can development of potential therapeutic targets (3).

Stroke thrombi are heterogeneous in composition, comprising varying proportions of fibrin, platelets, red blood cells (RBCs), leukocytes, and neutrophil extracellular traps (NETs) (3). These components reflect different pathophysiological processes and local hemodynamic conditions. For instance, thrombi formed under low-shear or static flow conditions are typically fibrin-rich and organized in structure, as often observed in cardiac-origin thrombi associated with atrial fibrillation (4). In contrast, thrombi formed under high-shear stress—such as those on valvular leaflets—tend to be platelet-rich and may indicate nonbacterial thrombotic endocarditis in the context of malignancy (5, 6). Thrombi formed under stasis or reduced flow conditions typically contain a higher proportion of RBCs due to erythrocyte entrapment within fibrin networks (2, 7–9).

The histological characteristics of stroke thrombi provide insights into their origin and inform tailored EVT strategies as well as the development of targeted thrombolytic agents (3). For example, RBC-rich thrombi are more susceptible to fragmentation during EVT, while platelet-rich thrombi are typically stiffer—both characteristics that may complicate retrieval and require tailored strategies during the procedure (10, 11). Recent studies have also identified the critical role of neutrophils and NETs in thrombus formation, structural stability, and resistance to reperfusion therapy, highlighting potential targets for future therapeutic strategies (3).

Despite the clinical importance of thrombus characteristics, the relationship between thrombus histological features and thrombus volume in acute ischemic stroke remains poorly understood. Thrombus volume itself is a key factor associated with higher stroke severity, larger infarct size, and lower recanalization rates following EVT (12, 13). As thrombi grow, the distribution and proportions of their components may dynamically change due to varying blood flow conditions, coagulation factors, or local hemorheology, potentially influencing clinical outcomes and guiding EVT strategies. In this study, we investigated whether thrombus components and spatial distribution patterns differed according to thrombus volume assessed in thin-section non-contrast computed tomography (NCCT). We also determined the association between clinical and radiological outcomes and volume-related thrombus characteristics.

## 2 Materials and methods

### 2.1 Patients

This retrospective analysis was based on data from a prospective nationwide multi-center registry, the Specialized Multi-center Attributed Registry of Stroke-CLOT (SMART-CLOT). The SMART-CLOT is a prospective registry that includes data of patients from 13 centers in South Korea who experienced acute consecutive ischemic strokes and underwent EVT for LVO. During hospitalization at each study hospital, all patients underwent a comprehensive assessment that included a review of their medical history, blood tests, CT, magnetic resonance imaging, carotid ultrasound, transcranial Doppler, 12-lead electrocardiography, echocardiography, and Holter monitoring or continuous electrocardiogram monitoring. Stroke severity was also evaluated using the National Institutes of Health Stroke Scale (NIHSS). Reperfusion therapy was performed according to guideline-based protocols and the attending physician’s discretion, depending on the patient’s clinical status. Written informed consent was obtained from prospectively enrolled patients or their next of kin. The institutional review board approved the registry of each participating hospital.

This study included consecutive patients who underwent EVT and whose thrombi were obtained between July 2017 and July 2023. In addition, to reliably measure the volume of the culprit thrombus, only 235 patients who underwent thin-section (1 or 1.25 mm) NCCT using the same protocols before EVT at the Severance Stroke Center were included. This study was approved by the Institutional Review Board of the Yonsei University College of Medicine (approval number: 2023-2440-001).

### 2.2 Assessment of thrombus volume

For patients with intracranial LVO, thrombus volume and density (Hounsfield units) were measured using baseline thin-section NCCT with a semi-automatic three-dimensional software (Xelis; Infinitt, Seoul, Korea), as described previously (14). Pixel segmentation suggested that the thrombus area should be blue at the threshold between 50 and 100 Hounsfield units. The region of interest for the thrombus was determined by clicking on any portion of the target area. Thereafter, automatic pixel dilation and growth of a region to the margin at a threshold between 40 and 100 Hounsfield units were performed by simply clicking the “dilate” icon. Subsequently, the thrombus volume was automatically measured and presented on a screen. Two stroke neurologists (Y.D.K. and J.Y.) who were not aware of any clinical information measured the thrombus volume. Given the excellent inter-rater agreement (intraclass correlation coefficient [ICC], 0.982; 95% confidence interval [CI], 0.970–0.989; *p* < 0.001), the measurements obtained by J.Y. were used for the analysis.

### 2.3 Thrombus preparation and immunohistochemistry

Thrombi retrieved from the EVT were promptly preserved in 4% paraformaldehyde, embedded in paraffin blocks, and stored. The fixed specimens were sectioned into 3 μm-thick slices, deparaffinized using xylene, and rehydrated using graded ethanol. For immunohistochemistry, the following primary antibodies were used: monoclonal anti-CD42b (ab134087, 1:100; Abcam, Cambridge, UK) for platelets, rabbit polyclonal anti-fibrinogen (ab34269, 1:200; Abcam) for fibrin/fibrinogen, and rabbit monoclonal anti-glycophorin A (ab129024, 1:400; Abcam) for RBCs. Antigen retrieval was performed using the IHC-Tek epitope retrieval solution and a steamer (IHC World, Woodstock, MD, USA) for all antibodies except for anti-CD42b.

The 3 μm-thick tissue sections were incubated overnight at 4°C. Secondary antibody reactions were performed using an avidin/biotin/horseradish peroxidase complex (Vector Laboratories Ltd., Peterborough, Cambridgeshire, UK). Positive signals were visualized using 3,3′-diaminobenzidine.

After counterstaining with hematoxylin, the slides were sealed using Permount mounting medium (Fisher Scientific, Fair Lawn, NJ, USA). One slide was prepared per stain for each patient. Images of stained thrombi were obtained using a digital scanner (Aperio AT2; Leica Biosystems, Wetzlar, Germany). The scanned images were analyzed using Automated Region-of-interest based Image Analysis (ARIA) for automated composition analysis (15). The fractions of each component, including RBCs, platelets, and fibrin, were calculated as the percentage pixel density of the total thrombus area.

### 2.4 Composition analysis and assessment of immunohistochemistry patterns

**Figure 1:**
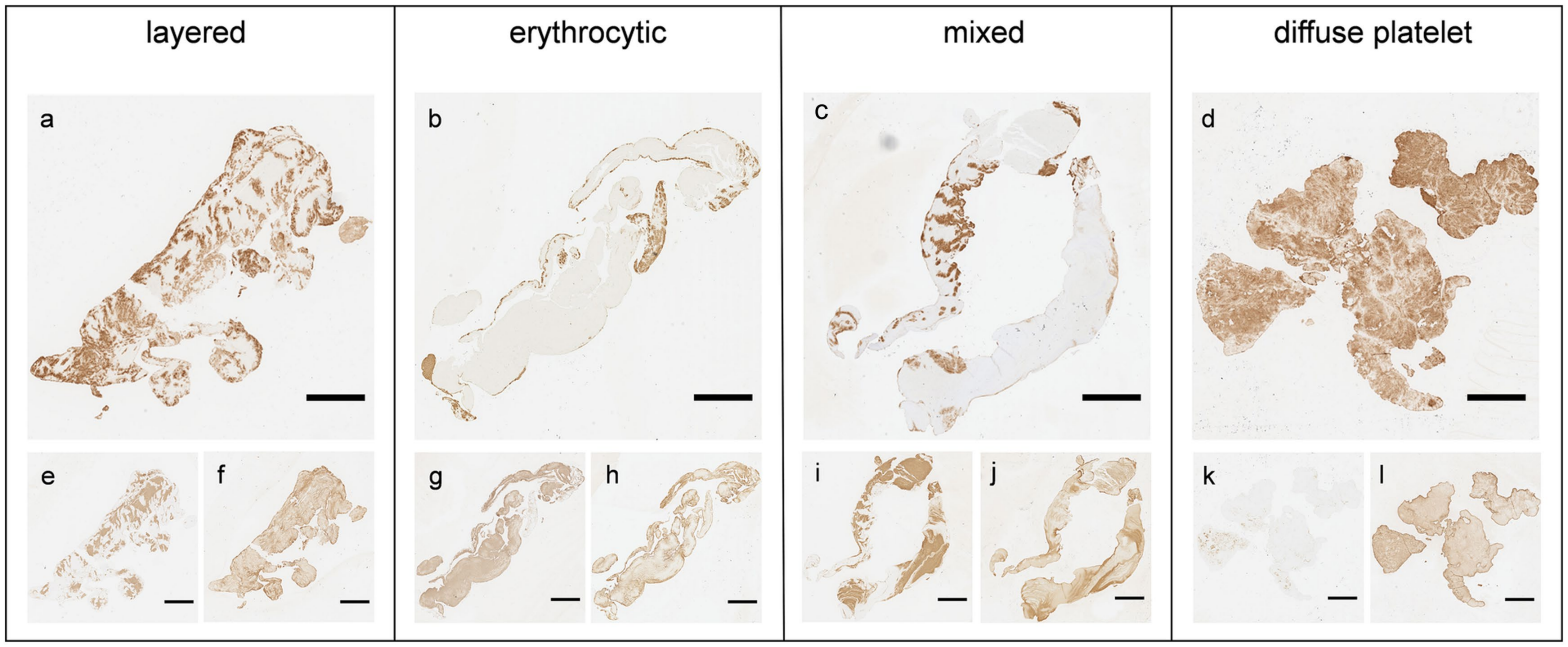
Four distribution patterns of thrombus based on immunohistochemical staining. Four representative thrombi were observed for each immunohistological pattern. **(a–d)** Platelets (anti-CD42b); **(e, g, i, k)** red blood cells (anti-glycophorin A); **(f, h, j, l)** fibrin (anti-fibrinogen). Scale bar = 2 mm.

The extracted thrombi were characterized into four groups based on their overall distribution patterns of common components (especially platelets and RBCs) based on previous reports (Figure 1) (9, 16). The distribution pattern of the platelets and RBCs was distinctive: first, the platelets and RBCs were deposited alternatively deposited in a linear, layered pattern or clustered in spot-like formations within the thrombus (9, 16). In some thrombi, the platelets were located predominantly at the periphery, filled with densely packed RBCs, which represented the erythrocytic pattern. They were similar the erythrocytic-type thrombi (red clot) reported in previous studies (9, 16). Specimens showing both peripherally located platelets and with packed RBCs, along with and internally alternating deposited platelets and RBC layers, were categorized under mixed pattern. Finally, thrombi, which is filled with platelets without any specific pattern were classified under diffuse platelet pattern.

### 2.5 Outcome measures

Stroke neurologists and research nurses regularly contacted the patients or their caregivers during follow-up sessions via regular face-to-face visits or telephone interviews, with or without a medical chart review, to investigate the clinical outcomes, including the modified Rankin score (mRS). The radiological outcomes, including the device pass number, modified Thrombolysis in Cerebral Infarction (TICI) grade, symptomatic intracranial hemorrhage (ICH), and procedure time, were also assessed. The first-pass effect was defined as near-complete or complete recanalization (TICI 2c or TICI 3) after the first pass of the device. The procedure time was defined as the interval (min) from the femoral puncture to the first achievement of a TICI of 2b or 3. In addition, we determined the number of fragmented thrombi during the procedure, which was defined as the number of newly apparent occlusions in the downstream vessel after recanalization of the culprit lesion.

### 2.6 Statistical analysis

Data are presented as the mean ± standard deviation (SD), median (interquartile range), or percentage (%), as appropriate. Continuous variables were compared using Student’s *t*-tests or analysis of variance, while categorical variables were compared using the Chi-square or Fisher’s exact tests, as appropriate. Correlations between thrombus volume and proportion of thrombus components were investigated using Pearson’s correlation analysis. Thrombus volume tertiles were used when comparing the distribution patterns according to thrombus volume. To identify independent factors associated with thrombus characteristics or outcomes, multiple linear or ordinal regression analyses were performed after adjusting for age, sex, and other variables with a *p*-value of < 0.1 in the univariable analyses. *p* < 0.05 was considered statistically significant. Statistical analyses were conducted using the R software package version 4.3.1^1^ or SPSS for Windows (version 27; SPSS, Chicago, IL, USA).

## 3 Results

### 3.1 Study population and baseline characteristics

Among 235 patients, we excluded the thrombi of 8 patients that were not detected on thin-section NCCT and those of 17 patients with no available immunohistochemical data. Finally, 210 patients were included in the study (Supplementary Figure 1).

The mean age of the 210 patients (± SD) was 73.0 ± 12.9 years, and 103 (49.0%) patients were men. The mean interval from stroke onset to baseline thin-section NCCT was 381.7 ± 480.2 min. Among the stroke mechanisms, cardioembolism (60.0%) was more common than large artery atherothrombosis (11.0%). Other baseline characteristics, including risk factors, medication history, laboratory variables, and treatment outcomes, are shown in Table 1.

**Table 1:** Baseline characteristics of the study group.

Variables	Statistics
Age, years	73.0 ± 12.9
Male, sex	103 (49.0)
Intravenous t-PA	64 (30.5)
Interval from stroke onset to CT performance, min	381.7 ± 480.2
Initial NIHSS score	13.0 (8.0, 18.0)
Risk factors	
Hypertension	146 (69.5)
Diabetes	69 (32.9)
Dyslipidemia	19 (9.0)
Atrial fibrillation	118 (56.2)
Previous stroke or TIA	45 (21.4)
Coronary artery disease	102 (66.2)
Peripheral arterial occlusive disease	26 (17.2)
Active cancer	23 (11.0)
Previous medication history	
Antiplatelets	38 (24.7)
Oral anticoagulants	31 (20.1)
Stroke mechanism	
Cardioembolism	126 (60)
Large artery atherothrombosis	23 (11.0)
Stroke of other determined etiology	7 (3.3.0)
Two or more causes identified	25 (11.9)
Negative evaluation	29 (13.8)
Occlusion site	
Middle cerebral artery	151 (71.9)
Terminus ICA	19 (9.0)
Cavernous ICA	11 (5.2)
Basilar artery	27 (12.9)
Posterior cerebral artery	2 (1.0)
Laboratory variables	
Hemoglobin, mmol/L	8.2 ± 2.1
White blood cell count, 10^9^/L	8223.3 ± 3584.1
Platelet count, 10^9^/L	208.8 ± 76.7
Fibrinogen, g/L	3.1 ± 0.9
Creatinine, μmol/L	86.5 ± 62.6
Albumin, g/L	40.4 ± 4.4
Treatment outcomes	
Procedure time, min^†^	51.7 ± 28.1
TICI grade^‡^	
0 or 1	7 (3.4)
2a	11 (5.2)
2b	50 (23.8)
2c	14 (6.7)
3	128 (61.0)
First pass effect^‡^	74 (35.2)
Number of device passage^‡^	
1	81 (38.6)
2	51 (24.3)
≥3	78 (37.1)
Number of fragmented thrombi^‡^	
0	112 (53.3)
1	51 (24.3)
2	18 (8.6)
3	16 (7.6)
4	9 (4.3)
5	4 (1.9)
Thrombus volume, mm^3^	43.7 (23.5, 74.5)

Values are number (%), median (interquartile range), or mean ± standard deviation. ^†^The results were obtained from 203 patients. ^‡^The result was evaluated in 210 patients. t-PA, tissue plasminogen activator; CT, computed tomography; NIHSS, National Institutes of Health Stroke Scale; TIA, transient ischemic attack; ICA, internal carotid artery; TICI, thrombolysis in cerebral infarction.

### 3.2 Association between thrombus volume and components

The most common occlusion site was the middle cerebral artery (*n* = 151 [71.9%]), followed by the distal internal carotid artery (ICA; terminal occlusion, *n* = 19 [9%]; cavernous occlusion, *n* = 11 [5.2%]), basilar artery (*n* = 27 [12.9%]), and posterior cerebral artery (*n* = 2 [1.0%]). The median (IQR) volume of the thrombus was 43.7 (23.5, 74.5) mm^3^ (Table 1). The median (IQR) thrombus volume based on the occlusion site was middle cerebral artery, 36.0 (22.0, 66.4) mm^3^; ICA-terminus occlusion, 77.6 (51.1, 118.4) mm^3^; ICA-cavernous occlusion, 66.9 (29.6, 122.8) mm^3^; basilar artery, 62.9 (43.6, 91.0) mm^3^; and posterior cerebral artery, 17.2 (14.6, 19.7) mm^3^ (Table 1).

**Figure 2:**
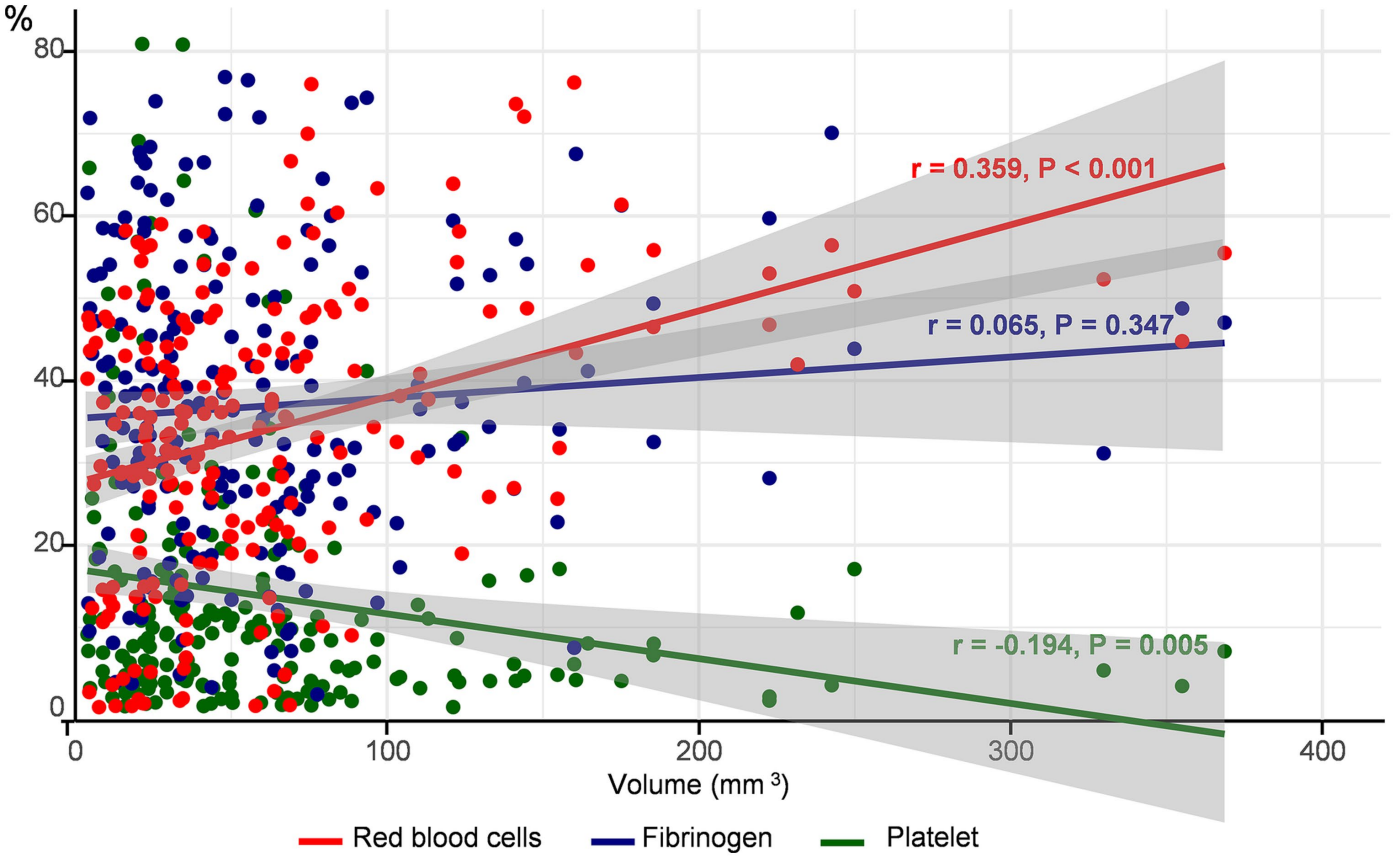
Correlation between thrombus volume and components. The proportion of each component of the thrombus according to thrombus volume is depicted. The red circles and lines represent red blood cells, blue represents platelets, and green represents fibrinogen.

The mean proportion (±SD) of the common components of the thrombus was 34.1 ± 15.5% for RBCs, 13.7 ± 15.5% for platelets, and 36.9 ± 17.9% for fibrinogen. The proportion of RBCs (anti-glycophorin A) increased (*r* = 0.359, *p* < 0.001), whereas that of platelets (anti-CD42b) decreased (*r* = −0.194, *p* = 0.005) with an increase in thrombus volume. However, fibrin (anti-fibrinogen) levels did not correlate with thrombus volume (*r* = 0.65, *p* = 0.347) (Figure 2). A positive association between the proportion of RBC and thrombus volume was observed, regardless of the stroke mechanism, when we determined the association between thrombus volume and composition across stroke mechanisms (Supplementary Figure 2).

### 3.3 Association between thrombus volume and thrombus patterns

The mixed pattern was the most common (*n* = 81 [38.6%]), followed by layered (*n* = 57 [27.1%]), erythrocytic (*n* = 47 [22.4%]), and diffuse platelet patterns (*n* = 25 [11.9%]). The mixed pattern was likely to survive the longest from stroke onset to CT (427.3 ± 503.8 min) and had the highest median NIHSS (15.0, interquartile range 9–20). The layered pattern was associated with coronary artery disease, atrial fibrillation, and cardioembolism, whereas the erythrocytic pattern was related to large artery atherothrombosis. More than half of the patients with diffuse platelet patterns had active cancer (*n* = 16 [64%]). The proportion of thrombus components differed according to the distribution patterns: the proportion of platelets was the highest in the diffuse platelet pattern and that of RBCs was the highest in the mixed pattern (all *p* < 0.05). However, the proportion of fibrinogen did not differ according to this pattern (Supplementary Table 1).

**Figure 3:**
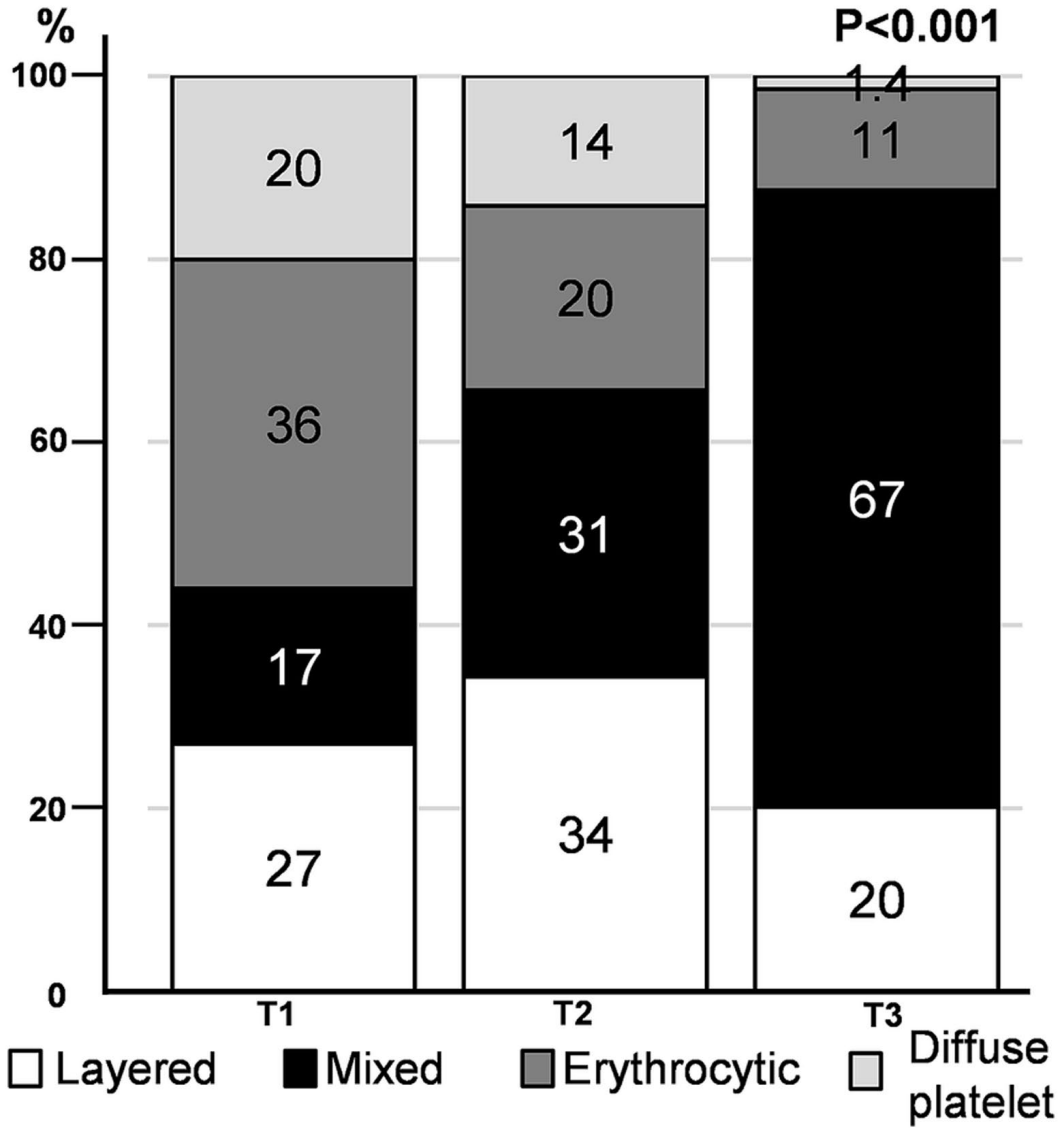
Immunohistochemistry pattern according to thrombus volume tertile. The proportion of distribution patterns in each thrombus volume tertile is depicted.

We found that the distribution pattern differed according to thrombus volume. The mixed pattern had the largest volume (median [IQR]) of thrombus (71.2 [41.2, 122.4] mm^3^), followed by layered (36.4 [23.5, 62.9] mm^3^), erythrocytic (29.3 [19.3, 48.7] mm^3^), and diffuse platelet (24.1 [20.0, 35.5] mm^3^) patterns (Supplementary Table 1). As the tertiles of thrombus volume increased, a mixed pattern was commonly observed, whereas an erythrocytic or diffuse platelet pattern was noted infrequently (*p* < 0.001) (Figure 3). When we divided the study groups based on the median volume (43.7 mm^3^) of the thrombus (smaller vs. larger thrombus), the mixed pattern was the most common in larger thrombi, regardless of the stroke mechanism (Supplementary Figure 3).

Additionally, thrombus volume was associated with serum hemoglobin level, initial NIHSS score, history of dyslipidemia, previous stroke or transient ischemic attack, and presence of active cancer (all *p* < 0.05), whereas it was not correlated with the interval from stroke onset to CT, use of tissue plasminogen activator or prior use of antithrombotic agents, and stroke mechanism. Multivariable analysis adjusting for age, sex, and significant variables in the univariable analysis revealed an independent and significant positive association between thrombus volume and the mixed pattern (*β* 21.04, SE 10.10, *p* = 0.038) compared to the layered pattern or proportion of RBCs (*β* 1.00, SE 0.27, *p* < 0.001) and an inverse association with the erythrocytic pattern (*β* − 29.78, SE 11.54, *p* = 0.011) compared to the layered pattern (Table 2).

**Table 2:** Factors related to analysis of thrombus volume.

Variables	Univariable analysis			Multivariable analysis		
	*B*	SE	*p*	*B*	SE	*p*
Age, year	−0.42	0.349	0.226	−0.588	0.326	0.073
Male, sex	14.24	8.93	0.112	11.689	8.02	0.147
Intravenous t-PA	−6.06	9.75	0.535			
Interval from stroke onset to CT perform, min	−0.0004	0.009	0.962			
Initial NIHSS score	1.32	0.643	0.041			
Risk factors						
Hypertension	−14.62	9.71	0.134			
Diabetes	2.21	9.56	0.817			
Dyslipidemia	−23.65	9.71	0.016	−19.54	8.63	0.025
Atrial fibrillation	11.76	9.02	0.194			
Previous stroke or TIA	22.66	10.83	0.038	21.8	9.68	0.025
Coronary artery diseases	−14.20	12.46	0.256			
Peripheral arterial occlusive disease	14.73	11.88	0.216			
Active cancer	−41.53	14.09	0.004	−8.94	17.31	0.606
Previous medication history						
Antiplatelets	−10.12	11.11	0.363			
Oral anticoagulants	−9.25	11.02	0.402			
Stroke mechanism						
Cardioembolism	−1.29	9.17	0.888			
Large artery atherothrombosis	−6.34	14.38	0.660			
Stroke of other determined etiology	−3.78	25.03	0.880			
Two or more causes identified	17.99	13.82	0.194			
Negative evaluation	−7.03	13.01	0.590			
Laboratory findings						
Hemoglobin, mmol/L	6	2.11	0.005	2.01	2.03	0.324
White blood cell count, 10^9^/L	0.0003	0.001	0.782			
Platelet count, 10^9^/L	−0.17	0.059	0.768			
Fibrinogen, g/L	4.04	5.04	0.424			
Creatinine, μmol/L	−0.007	0.072	0.925			
Albumin, g/L	1.84	1.01	0.069			
Treatment outcomes^†^						
Procedure, min	0.32	0.161	0.048			
TICI grade of 2c or 3	−1.68	9.6	0.861			
First-pass effect	−9.417	9.382	0.317			
Number of device passage	3.29	3.36	0.121			
Number of fragmented thrombi	16.225	3.36	<0.001			
Any cerebral hemorrhage	13.27	10.12	0.191			
Symptomatic cerebral hemorrhage	54.92	14.48	<0.001			
Stroke outcomes						
Modified Rankin score (mRS) at 3 months	−0.018	2.006	0.993			
Poor functional outcome (mRS ≥ 3) at 3 months	−1.66	8.99	0.854			
Mortality at 3 months	−0.589	12.06	0.961			
Microscopic components						
Platelets	−0.816	0.286	0.005	−0.250	0.370	0.507
Red blood cells	1.332	0.240	<0.001	1.00	0.270	<0.001
Fibrinogen	0.236	0.251	0.347			
Immunohistochemistry pattern						
Layered	Ref			Ref		
Mixed	33.16	10.49	0.002	21.04	10.10	0.038
Erythrocytic	−19.53	11.96	0.104	−29.78	11.54	0.011
Diffuse platelet	−28.92	14.56	0.048	−5.31	18.09	0.77

^†^Procedure time was assessed in 203 patients, whereas all the other variables relating to treatment outcomes were evaluated in 210 patients. B, B coefficient; SE, standard error; CT, computed tomography; NIHSS, National Institutes of Health Stroke Scale; t-PA, tissue plasminogen activator; TIA, transient ischemic attack; TICI, thrombolysis in cerebral infarction; mRS, modified Rankin score.

### 3.4 Outcomes according to thrombus volume

The number of fragmented thrombi, procedure time, and frequency of symptomatic ICH increased with thrombus volume (all *p* < 0.005). Poor functional outcome (indicating mRS ≥ 3) or mortality after 3 months from index stroke was not related to thrombus volume (Table 2). Univariable and multivariable ordinal regression analyses showed that the number of fragmented thrombi was independently associated with the proportion of RBCs and thrombus volume. However, the distribution pattern was not associated with the number of fragmented thrombi (Table 3; Supplementary Table 2). Other radiological, clinical outcomes were described at Supplementary Table 3.

**Table 3:** Ordinal multivariable analysis related to the number of fragmented thrombi.

Variables	*B*	SE	*p*
Age, year	0.001	0011	0.931
Male, sex	−0.081	0.276	0.771
Interval from stroke onset to CT perform, min	−0.001	0.000427	0.046
Intravenous t-PA	0.557	0.327	0.088
Thrombus volume, mm^3^	0.006	0.002	0.006
Microscopic components			
Red blood cells	0.018	0.009	0.049
Immunohistochemistry pattern			
Layered	−0.058	0.350	0.869
Mixed	Ref		
Erythrocytic	−0.120	0.386	0.757
Diffuse platelet	−0.124	0.561	0.825

CT, computed tomography; t-PA, tissue plasminogen activator.

## 4 Discussion

Using radiological and histological analyses, we detected an increase in the proportion of RBCs and a decrease in the proportion of platelets with an increase in thrombus volume in this cohort study. A larger thrombus was associated with a mixed pattern, whereas a smaller thrombus was related to an erythrocytic or diffuse platelet pattern. The proportion of RBCs and mixed patterns were independently associated with larger thrombi.

Incorporation of RBCs was a key component contributing to thrombus volume. This observation agrees with that in previous studies showing that large thrombi are more likely to be hyperdense on CT scans or appear as blooming artifacts on gradient echo magnetic resonance imaging (17, 18). Previous studies using human thrombus have shown fibrin to be the most abundant component (23–74%) (4, 19). However, the proportion of RBCs passively entrapped between fibrin networks might become notably larger during thrombus growth because the size of RBCs was larger than any other component. Moreover, previous studies have suggested that RBCs play a more active role in thrombosis than previously thought. For example, RBCs can promote platelet margination and activate or augment thrombin generation (20). Thrombus propagation could be mediated by the interaction between RBCs and the endothelium, platelets, and fibrin (20, 21). In addition, RBCs can be actively retained within thrombi by coagulation factor XIII(a)-mediated fibrin *α*-chain crosslinking (22). These findings suggest that RBCs may be deeply involved in thrombus growth.

A comparison between the distribution patterns and thrombus volume revealed that the distribution of common components (such as fibrin, platelets, and RBCs) can be influenced by flow conditions or shear stress at the thrombus formation sites during thrombus formation (3). We initially identified four distinctive thrombi distribution patterns that were partially modified versions of those mentioned in previous studies (9, 16). Among these patterns, the layered pattern with platelets/fibrin and RBCs appearing as variegated spots (lines of Zahn) typically forms under high shear stress in rapid flow conditions within the heart, aorta, or veins (2, 4, 7, 8). Conversely, the erythrocytic pattern, characterized by peripherally located platelets and centrally located RBCs, resembles a coagulation clot (red clot) and is usually formed under low shear stress conditions (3, 8, 23). This thrombus pattern is formed by a fibrin mesh entangling masses of RBCs under both turbulent and stagnant blood flow conditions, such as in venous pockets or post-stenotic recirculation areas (2, 7–9). In this study, we observed for the first-time thrombi simultaneously exhibiting both layered and erythrocytic patterns, suggesting that they may have formed under mixed hemodynamic conditions involving both rapid flow and turbulent or stagnant flow with varying flow rates. For example, the erythrocytic pattern might develop in post-stenotic areas following the formation of layered pattern thrombi due to high shear stress at the stenosis site (7, 8). Finally, a diffuse platelet pattern with dense platelets and sparse RBCs is typically observed in cancer-related thrombi or non-bacterial thrombotic endocarditis (6) (Supplementary Table 4).

We observed that thrombus volume was positively associated with mixed patterns, whereas an inverse relationship was observed between thrombus volume and erythrocytic or diffuse platelet patterns. Layered patterns are frequently found in cardioembolic stroke and erythrocytic patterns in large artery atherothrombosis (4), whereas mixed patterns are frequently observed across a range of stroke etiologies. As mentioned above, mixed patterns can form under both low and high shear stress conditions (7, 8), Considering that intracardiac conditions in cardioembolism or intra-arterial conditions around the stenotic segment in atherothrombosis are characterized by complex flow patterns that significantly enhance thrombosis (24), mixed patterns might reflect the multiple phases of thrombus growth. In addition, fresh thrombi can form locally due to blood stasis around the original thrombus (17). Components of the mixed pattern, especially RBCs, may accumulate around the initial occlusion site created by the original thrombus. In our analysis, the mixed pattern with the largest thrombus volume survived the longest, from stroke onset to CT imaging. Finally, the association between smaller thrombi and diffuse platelet pattern can be explained by the fact that the thrombi in cancer-associated strokes, which frequently exhibit this pattern, typically involve multiple small infarction lesions.

We found that the proportion of RBCs and thrombus volume were independent factors associated with the number of clot fragments. As the thrombus enlarged, the stent retrieval device was less likely to capture the entire thrombus segment. A large thrombus also has a larger friction area that resists the negative suction force applied to the proximal end of the thrombus (25). Compositional changes in thrombus according to its volume may affect clot fragmentation as RBCs, fibrin, and platelets exhibit different levels of stability. RBC aggregation via passive packing is less stable than adhesive binding through integrin α_2b_*β*_3_ to fibrinogen (11). Moreover, RBCs can modify fibrin structure, reduce its friction coefficient, and render the thrombus deformable, subsequently making it susceptible to fragmentation (26, 27). These findings suggest that thrombus volume and composition—particularly RBC-rich clots—may influence fragmentation risk during EVT. In such cases, the use of larger bore aspiration catheters, balloon-guide catheters, or adjunctive devices (e.g., distal access catheters or combined techniques) may improve procedural efficacy by enhancing thrombus capture and minimizing fragmentation.

This study has several strengths. Each component of the thrombus was identified using immunohistochemical staining, presenting a more specific and accurate composition. In addition, we compared the histological pattern and quantity of the thrombus components with thrombus volume. We used independent semi-automated software tools for quantifying thrombus volume on thin-slice non-contrast CT (14), and for analyzing histological composition based on immunohistochemistry (15). Both have demonstrated reliability and consistency in previous studies (14, 15).

This study has certain limitations. First, although we used reliable methods for measuring thrombus volume and its composition, thrombi can change even in the initial stages after a stroke (14). Secondly, as an exploratory study, our findings may have been influenced by events that occurred in thrombi prior to retrieval. In ischemic stroke, embolic thrombi are common, and small thrombi (less than 50 mm^3^) may include fragments of larger thrombi. This inclusion could dilute the correlation between thrombus volume and composition, potentially explaining the lower correlation coefficients observed in smaller thrombi (Supplementary Table 5). Additionally, during endovascular treatment, inevitable loss or alteration of thrombi may influence immunohistochemistry result, although we analyzed all the obtained thrombi. Third, the sample size of patients with large artery atherothrombosis in this study was limited. Fourth, our study did not find any clinical or procedural outcomes independently associated with thrombus volume or its microscopic changes, except for the number of fragmented thrombi during the procedure. This lack of significant associations may be attributed to not considering detailed EVT techniques or devices, which can affect EVT outcomes (28). However, since thrombus composition is closely related to the mechanical interactions with EVT devices, our findings may provide insights into selecting appropriate EVT strategies based on the volume of the occluding thrombus (10, 25). Lastly, although our study focused on thrombus volume quantified by thin-section non-contrast CT, radiologic features beyond volume—such as CT density or radiomic characteristics, which may better reflect thrombus composition—were not included in the present analysis. Future studies incorporating these imaging features may enhance non-invasive prediction of thrombus histology and support the development of tailored EVT strategies. Further studies integrating radiologic analyses of neuroimaging with histopathologic findings are warranted to enable non-invasive prediction of thrombus composition. Such efforts may ultimately support the development of tailored EVT strategies based on preprocedural thrombus characteristics.

## 5 Conclusion

RBC incorporation significantly contributed to the volumetric growth of thrombi in ischemic strokes and their susceptibility to fragmentation. In addition, the mixed pattern was commonly observed as the thrombus volume increased. Our findings emphasize that thrombus components and their distribution could differ with thrombus growth. These observations should be considered in the choice of EVT strategy to optimize EVT outcomes and in future research to determine the etiologic relevance of thrombus characteristics.

## Data Availability

The raw data supporting the conclusions of this article will be made available by the authors, without undue reservation.

## Footnotes

^1^ http://www.R-project.org
