# Supplementary material for: Thrombus composition and distribution patterns by thrombus volume in acute ischemic stroke
Source: Front Neurol. 2025 Aug 4;16:1619683. doi: 10.3389/fneur.2025.1619683 (PMC12358443; doi:10.3389/fneur.2025.1619683)
Supplement: Supplementary file 1 [file Table_1.docx]

**Supplemental Materials**

**Table of Contents**

**Supplemental Figure 1. Patient selection diagram**

**Supplemental Figure 2. Correlation between thrombus volume and components according to stroke mechanisms**

**Supplemental Figure 3. Comparison of the distribution patterns by stroke mechanisms among patients with (A) smaller thrombus (<63.2 mm3) and (B) larger thrombus (≥63.2 mm3)**

**Supplemental Table 1. Baseline characteristics stratified by thrombus pattern**

**Supplemental Table 2. Univariable analysis for the number of fragmented thrombi**

**Supplemental Table 3. Radiologic and clinical outcomes according to thrombus volume and histological features associated with thrombus volume**

**Supplemental Table 4. Morphologic, Hemodynamic, and Etiologic Characteristics of Thrombi According to Immunohistochemical Distribution Pattern**

**Supplemental Table 5. Correlation analysis of thrombus volume and microscopic components stratified by thrombus volume (50mm3)**

**Supplemental Figure 1. Patient selection diagram**


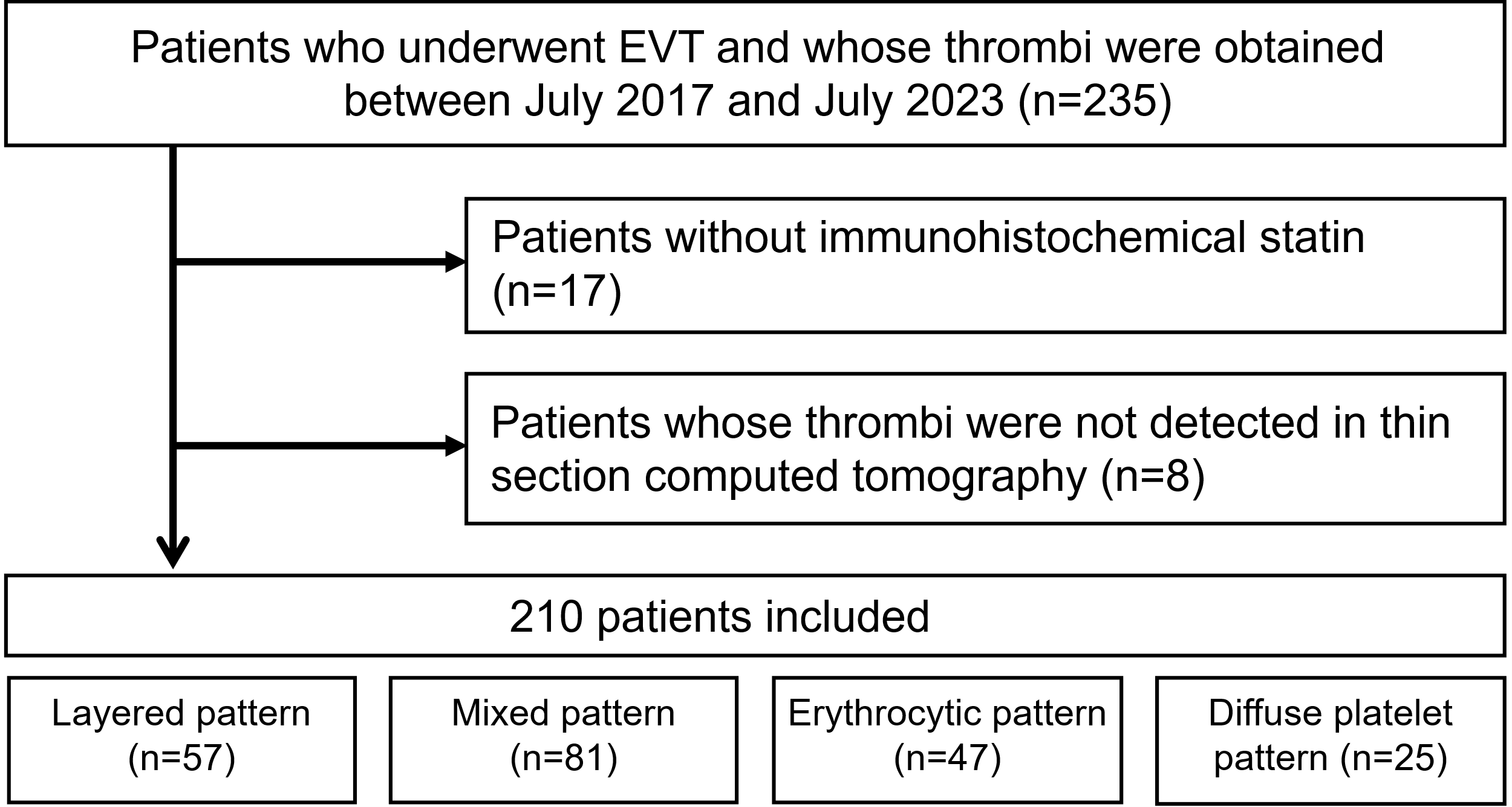


Abbreviations: NCCT, non-contrast computed tomography; EVT, Endovascular therapy

**Supplemental Figure 2. Correlation between thrombus volume and components according to stroke mechanisms**

**
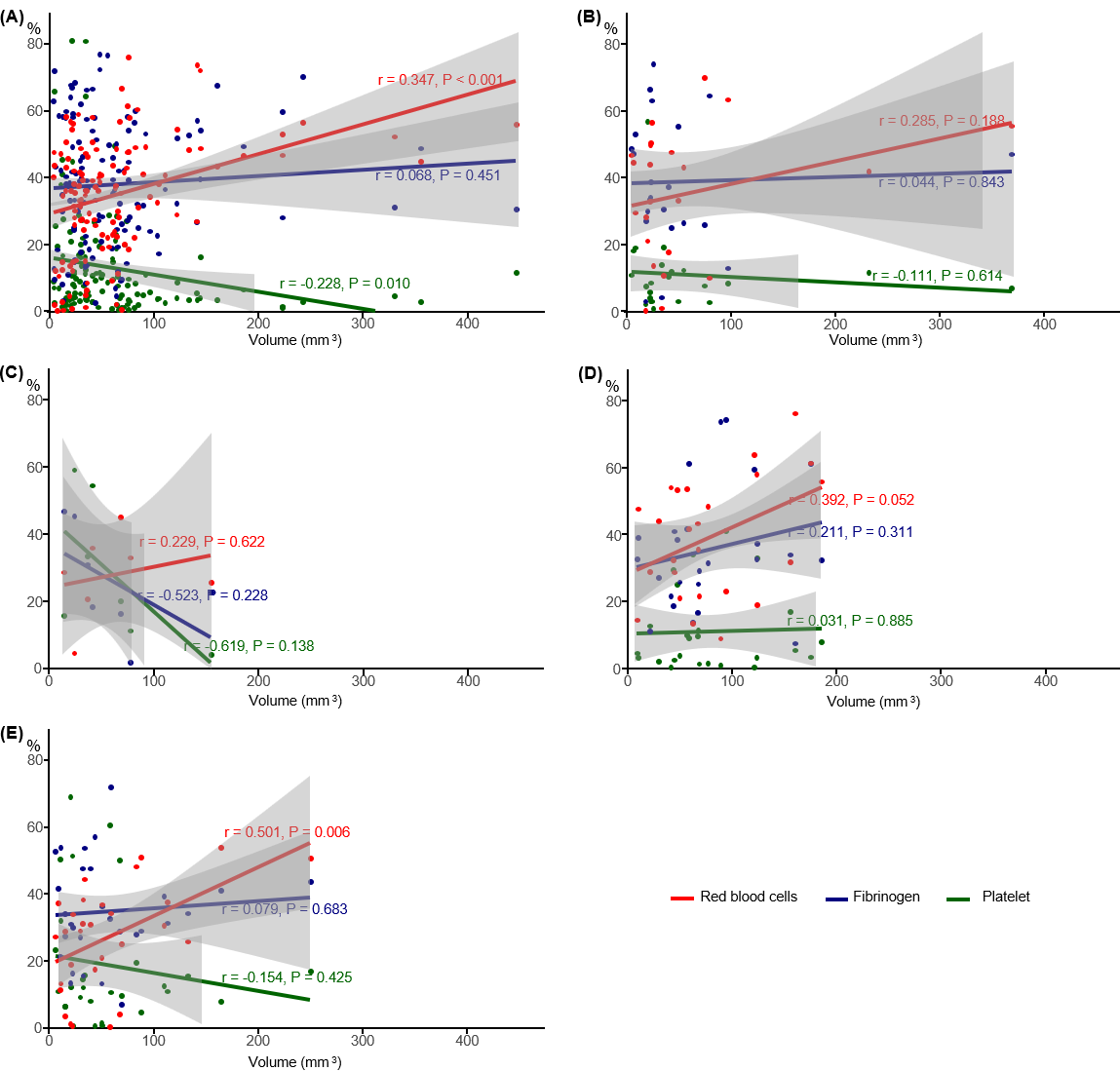
**

(A) Cardioembolism (B) Large artery atherothrombosis (C) Stroke of other determined etiology (D) Two or more causes identified (E) Negative evaluation

**Supplemental Figure 3. Comparison of the distribution patterns by stroke mechanisms among patients with (A) smaller thrombus (<63.2 mm^3^) and (B) larger thrombus (≥63.2 mm^3^)**

**
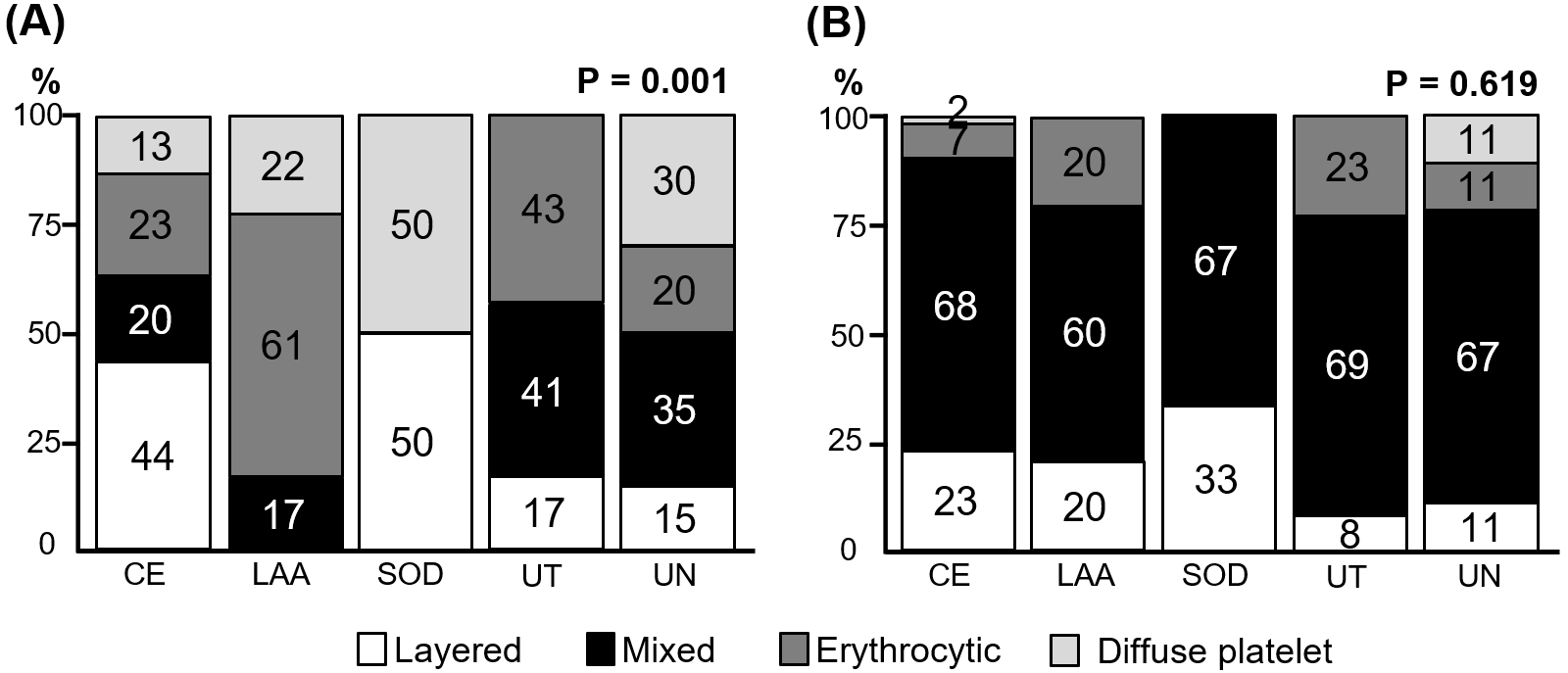
**

Abbreviations: CE, Cardioembolism; LAA, Large artery atherothrombosis; SOD, Stroke of other determined etiology; UT, Two or more causes identified; UN, Negative evaluation.

**Supplemental Table 1. Baseline characteristics stratified by thrombus pattern**

|  | **Immunohistochemistry pattern** | | | | P |
| --- | --- | --- | --- | --- | --- |
|  | Layered  (n = 57) | Mixed  (n = 81) | Erythrocytic  (n = 47) | Diffuse platelet  (n = 25) |  |
| Age, years | 73.6 ± 13.7 | 74.4 ± 12.5 | 74.7 ± 10.8 | 64.0 ± 12.5 | **0.003** |
| Male, sex | 28 (49.1) | 37 (45.7) | 22 (46.8) | 16 (64.0) | 0.4 |
| Intravenous t-PA | 28 (49.1) | 24 (29.6) | 7 (14.9) | 5 (20.0) | **0.001** |
| Interval from stroke onset to CT, min | 355.0 ± 590.9 | 427.3 ± 503.8 | 403.1 ± 349.1 | 254.8 ± 295.2 | **0.026** |
| Initial NIHSS score, median (interquartile range) | 14.0 (10.0 - 18.0) | 15.0 (9.0 - 20.0) | 11.0 (5.5 - 15.5) | 11.0 (6.0 - 16.0) | **0.006** |
| **Risk factors** |  |  |  |  |  |
| Hypertension | 41 (71.9) | 58 (71.6) | 31 (66.0) | 16 (64.0) | 0.8 |
| Diabetes | 17 (29.8) | 32 (39.5) | 13 (27.7) | 7 (28.0) | 0.4 |
| Dyslipidemia | 21 (36.8) | 23 (28.4) | 11 (23.4) | 7 (28.0) | 0.5 |
| Atrial fibrillation | 9 (68.4) | 46 (56.8) | 27 (57.4) | 6 (24.0) | **0.003** |
| Previous stroke or TIA | 11 (19.3) | 20 (24.7) | 11 (23.4) | 3 (12.0) | 0.6 |
| Coronary artery disease | 49 (86.0) | 58 (71.6) | 30 (63.8) | 10 (40.0) | **<0.001** |
| Peripheral arterial occlusive disease | 11 (19.3) | 18 (22.2) | 7 (14.9) | 0 (0.0) | **0.038** |
| Active cancer | 2 (3.5) | 2 (2.5) | 3 (6.4) | 16 (64.0) | **<0.001** |
| **Previous medication history** |  |  |  |  |  |
| Previous antiplatelets | 13 (22.8) | 18 (22.2) | 9 (19.1) | 3 (12.0) | 0.7 |
| Previous oral anticoagulants | 7 (12.3) | 14 (17.3) | 15 (31.9) | 8 (32.0) | **0.037** |
| **Stroke mechanism** |  |  |  |  | **<0.001** |
| Cardioembolism | 46 (80.7) | 46 (56.8) | 22 (46.8) | 12 (48.0) |  |
| Large artery atherothrombosis | 1 (1.8) | 6 (7.4) | 12 (25.5) | 4 (16.0) |  |
| Stroke of other determined etiology | 3 (5.3) | 2 (2.5) | 0 (0.0) | 2 (8.0) |  |
| Two or more causes identified | 3 (5.3) | 14 (17.3) | 8 (17.0) | 0 (0.0) |  |
| Negative evaluation | 4 (7.0) | 13 (16.0) | 5 (10.6) | 7 (28.0) |  |
| **Occlusion site** |  |  |  |  | 0.133 |
| Middle cerebral artery | 43 (75.4) | 50 (61.7) | 37 (78.7) | 21 (84.0) |  |
| Terminus ICA | 4 (7.0) | 10 (12.3) | 2 (4.3) | 3 (12.0) |  |
| Cavernous ICA | 3 (5.3) | 3 (3.7) | 4 (8.5) | 1 (4.0) |  |
| Basilar artery | 6 (10.5) | 17 (21.0) | 4 (8.5) | 0 (0.0) |  |
| Posterior cerebral artery | 1 (1.8) | 1 (1.2) | 0 (0.0) | 0 (0.0) |  |
| **Laboratory variables** |  |  |  |  |  |
| Hemoglobin, mmol/L | 8.2 ± 1.3 | 8.6 ± 2.9 | 8.0 ± 1.2 | 7.7 ± 1.5 | 0.4 |
| White blood cell count, 10^9^/L | 9,004.9 ± 5,192.3 | 7,766.8 ± 2,382.9 | 8,047.4 ± 2,899.3 | 8,251.2 ± 3,421.4 | 0.7 |
| Platelet count, 10^9^/L | 218.6 ± 82.2 | 212.4 ± 67.2 | 217.4 ± 68.7 | 159.0 ± 91.5 | **0.013** |
| Fibrinogen, g/L | 3.1 ± 1.0 | 3.1 ± 0.8 | 3.3 ± 0.9 | 2.7 ± 0.9 | 0.072 |
| Creatinine, µmol/L | 83.9 ± 30.5 | 82.7 ± 51.6 | 99.0 ± 107.5 | 80.8 ± 22.9 | 0.5 |
| Albumin, g/L | 39.6 ± 3.7 | 41.3 ± 3.8 | 40.3 ± 4.9 | 39.4 ± 6.3 | 0.068 |
| Procedure time, min^†^ | 51.8 ± 25.9 | 54.4 ± 28.4 | 53.7 ± 32.0 | 39.1 ± 22.2 | 0.064 |
| TICI grade^‡^ |  |  |  |  |  |
| 0 or 1 | 0 (0.0) | 0 (0.0) | 4 (8.5) | 3 (12.0) |  |
| 2a | 2 (3.5) | 4 (4.9) | 4 (8.5) | 1 (4.0) |  |
| 2b | 10 (17.5) | 19 (23.5) | 13 (27.7) | 8 (32.0) |  |
| 2c | 2 (3.5) | 6 (7.4) | 5 (10.6) | 1 (4.0) |  |
| 3 | 43 (75.4) | 52 (64.2) | 21 (44.7) | 12 (48.0) |  |
| First pass effect^‡^ | 20 (35.1) | 29 (35.8) | 17 (36.2) | 8 (32.0) | >0.9 |
| Number of device passage^‡^ |  |  |  |  | 0.4 |
| 1 | 21 (36.8) | 32 (39.5) | 20 (42.6) | 8 (32.0) |  |
| 2 | 19 (33.3) | 15 (18.5) | 12 (25.5) | 5 (20.0) |  |
| ≥ 3 | 17 (29.8) | 34 (42.0) | 15 (31.9) | 12 (48.0) |  |
| Number of fragmentated thrombi^‡^ |  |  |  |  | 0.759 |
| 0 | 29 (50.9) | 39 (48.1) | 27 (57.4) | 17 (68.0) |  |
| 1 | 16 (28.1) | 18 (22.2) | 12 (25.5) | 5 (20.0) |  |
| 2 | 5 (8.8) | 7 (8.6) | 5 (10.6) | 1 (4.0) |  |
| 3 | 3 (5.3) | 11 (13.6) | 1 (2.1) | 1 (4.0) |  |
| 4 | 3 (5.3) | 3 (3.7) | 2 (4.3) | 1 (4.0) |  |
| 5 | 1 (1.8) | 3 (3.7) | 0 (0.0) | 0 (0.0) |  |
| Thrombus volume, mm^3^ | 36.4 (23.5, 62.9) | 71.2 (41.2, 122.4) | 29.3 (19.3, 48.7) | 24.1 (20.0, 35.5) | **<0.001** |
| **Microscopic component** |  |  |  |  |  |
| Platelets | 14.1 ± 11.0 | 9.0 ± 7.2 | 7.1 ± 8.7 | 40.7 ± 23.6 | **<0.001** |
| Red blood cell | 31.7 ± 15.3 | 39.7 ± 14.7 | 38.3 ± 17.9 | 13.5 ± 13.6 | **<0.001** |
| Fibrinogen | 38.2 ± 17.3 | 36.3 ± 16.4 | 37.7 ± 19.5 | 34.2 ± 21.5 | 0.7 |

Note: ^†^Assessed in 203 patients; ^‡^Assessed in 210 patients.

Abbreviations: t-PA, tissue-plasminogen activator; CT, computed tomography; NIHSS, National Institutes of Health Stroke Scale; TIA, transient ischemic attack; ICA, internal carotid artery; TICI, thrombolysis in cerebral infarction, NBTE = nonbacterial thrombotic endocarditis.

Values are number (%), median (interquartile range) or mean ± standard deviation.

**Supplemental Table 2.** **Univariable analysis for the number of fragmented thrombi**

|  | B | SE | P |
| --- | --- | --- | --- |
| Age | -0.001 | 0.01 | 0.901 |
| Male, sex | 0.033 | 0.262 | 0.899 |
| Intravenous t-PA | 0.76 | 0.282 | **0.007** |
| Interval from stroke onset to CT perform, min | -0.001 | 0.000381 | **0.011** |
| Initial NIHSS score | 0.022 | 0.019 | 0.238 |
| **Risk factors** |  |  |  |
| Hypertension | -0.486 | 0.281 | 0.083 |
| Diabetes | -0.2 | 0.281 | 0.478 |
| Dyslipidemia | -0.373 | 0.294 | 0.205 |
| Atrial fibrillation | 0.363 | 0.267 | 0.173 |
| Previous stroke or TIA | -0.035 | 0.32 | 0.914 |
| Coronary artery disease | -0.15 | 0.369 | 0.683 |
| Peripheral arterial occlusive disease | -0.13 | 0.351 | 0.711 |
| Active cancer | -0.37 | 0.438 | 0.394 |
| **Previous medication history** |  |  |  |
| Previous antiplatelets | -0.50 | 0.34 | 0.141 |
| Previous oral anticoagulants | 0.19 | 0.318 | 0.551 |
| **Stroke mechanism** |  |  |  |
| Cardioembolism | 0.151 | 0.268 | 0.575 |
| Large artery atherothrombosis | -0.127 | 0.425 | 0.766 |
| Stroke of other determined etiology | 0.551 | 0.699 | 0.430 |
| Two or more causes identified | -0.735 | 0.446 | 0.099 |
| Negative evaluation | 0.240 | 0.373 | 0.519 |
| **Laboratory variables** |  |  |  |
| Hemoglobin, mmol/L | -0.018 | 0.064 | 0.774 |
| White blood cell count, 10^9^/L | -1.95E-05 | 3.81E-05 | 0.609 |
| Platelet count, 10^9^/L | 0.002 | 0.002 | 0.24 |
| Fibrinogen, g/L | -0.002 | 0.147 | 0.992 |
| Creatinine, µmol/L | -0.000497 | 0.002152 | 0.817 |
| Albumin, g/L | -0.002 | 0.03 | 0.951 |
| Thrombus volume, mm^3^ | 0.008 | 0.002 | **<0.001** |
| **Microscopic component** |  |  |  |
| Platelets | -0.012 | 0.009 | 0.19 |
| Red blood cells | 0.026 | 0.008 | **0.001** |
| Fibrinogen | -0.001 | 0.007 | 0.865 |
| **Immunohistochemistry pattern** |  |  |  |
| Layered | -0.240 | 0.323 | 0.475 |
| Mixed | Ref |  |  |
| Erythrocytic | -0.519 | 0.352 | 0.140 |
| Diffuse platelet | -.0925 | 0.470 | **0.049** |

Abbreviations: t-PA, tissue-plasminogen activator; NIHSS, National Institutes of Health Stroke Scale; CT, computed tomography; TIA, transient ischemic attack

**Supplemental Table 3. Radiologic and clinical outcomes according to thrombus volume and histological features associated with thrombus volume**

|  | First pass effect | Successful recanalization | Procedure time | the number of fragmented thrombi | Favorable outcome | Mortality |
| --- | --- | --- | --- | --- | --- | --- |
| Thrombus volume, mm^3^ | 1.00 (0.99,1.00) | 1.01 (1.00, 1.03) | 0.05 (-0.01, 0.12) | **0.01 (0.00, 0.01)** | 1.00 (1.00, 1.00) | 1.00 (0.99, 1.01) |
| **Microscopic components** |  |  |  |  |  |  |
| Red blood cells | 1.00 (0.98, 1.01) | 0.98 (0.95, 1.01) | 0.19 (-0.04, 0.42) | **0.02 (0.01, 0.03)** | **1.02 (1.00, 1.04)*** | **0.97 (0.95, 0.99)*** |
| **Immunohistochemistry pattern** |  |  |  |  |  |  |
| Layered | 0.98 (0.49, 1.93) | 1.43 (0.27, 10.6) | -2.7 (-13, 7.2) | -0.22 (-0.65, 0.21) | 1.06 (0.54, 2.10) | 0.67 (0.24, 1.74) |
| Mixed | Ref. | Ref. | Ref. | Ref. | Ref. | Ref. |
| Erythrocytic | 1.10 (0.53, 2.27) | 0.35 (0.09, 1.31) | 3.2 (-7.4, 14) | -0.43 (-0.89, 0.02) | 1.65 (0.80, 3.47) | 0.84 (0.30, 2.19) |
| Diffuse platelet | 0.83 (0.33, 2.06) | 0.38 (0.08, 2.05) | 15 (-28, -2.2) | **0.58 (-1.1, -0.01)** | 0.58 (0.22, 1.43) | 1.86 (0.63, 5.21) |

Values are presented as odds ratio (OR, 95% CI) or regression coefficient (B, 95% CI), as appropriate.

Bold indicates p < 0.05.

*Not significant in multivariate analysis.

CT, computed tomography; tPA, tissue plasminogen activator; EVT, endovascular therapy.

**Supplemental Table 4.** **Morphologic, Hemodynamic, and Etiologic Characteristics of Thrombi According to Immunohistochemical Distribution Pattern**

|  | **Immunohistochemistry pattern** | | |  |
| --- | --- | --- | --- | --- |
|  | Layered | Mixed | Erythrocytic | Diffuse platelet |
| Morphologic characteristics | Variegated spot, lines of Zahn | Both layered and erythrocytic | Coagulation (red) clot | Dense platelets and sparse RBC |
| Local hemorheology | High shear, rapid flow | Both high and low flow | Low shear, slow flow | High shear, very rapid flow |
| Suggested origin | Cardioembolic more likely (Left atrial appendage) | Cardioembolic or atheroembolic | Atheroembolic (post-stenotic recirculation area), Venous pocket | Cancer (NBTE on the cardiac valves) |

RBC, red blood cell; NBTE, nonbacterial thrombotic endocarditis

**Supplemental Table 5. Correlation analysis of thrombus volume and microscopic components stratified by thrombus volume (50mm^3^**)

| **Thrombi smaller than 50 mm^3^ (n = 117)** | **r** | **p** |
| --- | --- | --- |
| Platelets | -0.107 | 0.25 |
| Red blood cells | 0.174 | 0.060 |
| Fibrinogen | -0.049 | 0.60 |
| **Thrombi larger than 50 mm^3^ (n = 93)** | r | p |
| Platelets | -0.164 | 0.12 |
| Red blood cells | 0.358 | <0.001 |
| Fibrinogen | 0.182 | 0.08 |
